# Public health round-up

**DOI:** 10.2471/BLT.16.011116

**Published:** 2016-11-01

**Respect and better working conditions for midwives**

A midwife in the Russian Federation examines a newborn baby. Midwives deliver the care throughout pregnancy and childbirth that helps to prevent maternal and newborn deaths. But according to a recent report prepared by the World Health Organization (WHO) with the International Confederation of Midwives and the White Ribbon Alliance, midwives often face discrimination, lack of respect and poor working conditions that hinder their ability to provide quality care. Read the full story in: *Midwives voices, midwives realities report 2016, *available on:

http://www.who.int/maternal_child_adolescent/documents/midwives-voices-realities

**Figure:**
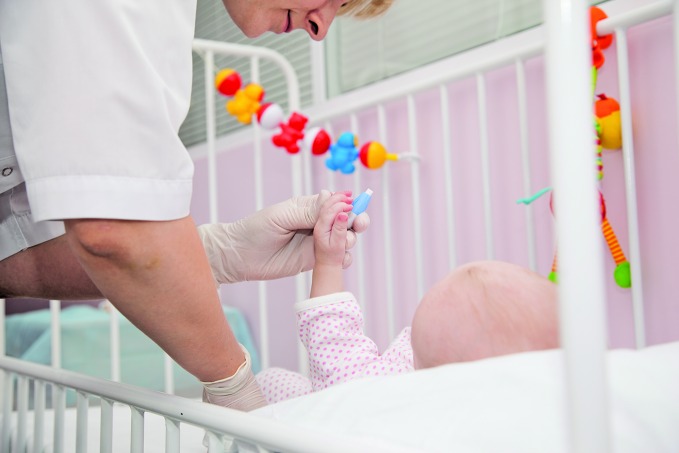
WHO /Sergey Volkov

## WHO election 

Six candidates for the election of the next WHO Director-General will present their vision to representatives of WHO Member States and Associate Members at a candidates’ forum this month.

The six candidates proposed by their respective governments: are Tedros Adhanom Ghebreyesus (Ethiopia), Flavia Bustreo (Italy), Philippe Douste-Blazy (France), David Nabarro (United Kingdom of Great Britain and Northern Ireland), Sania Nishtar (Pakistan) and Miklós Szócska (Hungary).

Each candidate will be required to do a one-hour interview with representatives of WHO Member States and Associate Members, comprising a presentation and a question-an-answer session. 

The candidates’ forum is part of the new process for the election of the WHO Director-General launched this year and takes place on 1–2 November at headquarters in Geneva. It will be webcast through the WHO website in all official languages for the public.

In January 2017, WHO’s Executive Board will screen all the candidatures and shortlist five of them based on one or more secret ballots.

Members of the Executive Board will then interview the shortlisted candidates and nominate three of them to go forward to the World Health Assembly (WHA) in May 2017 for the final round of voting. Previously, only one nomination was submitted by WHO’s Executive Board to the World Health Assembly.

The candidates’ forum follows a web forum held in October in which representatives from Member States asked candidates questions on a password-protected platform.

Dr Margaret Chan, who was first elected Director-General in 2006, will complete her second term on 30 June 2017 and the new Director-General will take office the next day.

http://apps.who.int/gb/ep/

## ICD-11 released for consultation

The draft 11th version of the *International statistical classification of diseases and related health problems* (ICD) was released last month by WHO for comments from Member States.

The ICD is the standard diagnostic reference book, published online and in print, for epidemiology, health management and clinical practice.

The current version, *ICD-10*, is in the final stages of being updated to reflect the latest advances in the health sciences and medical practice.

The draft *ICD-11* was released for consultation during a high-level ICD-11 Revision Conference for Member States hosted by the WHO Collaborating Centre WHO-Family of International Classifications in Japan.

The consultation period with Member States ends on 31 March 2017. Following any subsequent revisions, *ICD-11* is due to be published in 2018.

ICD contains codes for thousands of diseases and health conditions and is used by health workers, researchers, health information managers and coders, health information technology workers, policy-makers, insurers and patient organizations.

The current version, *ICD-10*, has been translated into 43 languages and is being used by all WHO Member States. Most countries (117) use the system to report mortality data, a primary indicator of health status.

*ICD-10* was endorsed at the 43rd World Health Assembly in 1990 and came into use in Member States from 1994.

http://www.who.int/classifications/icd/

## Fiscal measures for health

A new WHO report advises countries on how to reduce or prevent noncommunicable diseases such as obesity, diabetes and heart disease, by taking measures such as taxing sugar-sweetened beverages and subsidizing fruit and vegetables.

The report entitled *Fiscal policies for Diet and Prevention of Noncommunicable Diseases (NCDs)* calls on countries to consider appropriately designed measures, including taxes and subsidies, to give consumers incentives to choose healthy foods and drinks.

The report, released last month, is based on the findings of a technical meeting held last year at WHO headquarters to address requests from WHO’s Member States for guidance on how to design fiscal policies on diet and nutrition to improve their population’s health.

Experts at the meeting agreed that there is evidence that appropriately designed taxes on sugar sweetened beverages result in lower consumption of such drinks and would help to reduce obesity, type 2 diabetes and tooth decay. 

Such policies should, therefore, be considered as a key component of a comprehensive strategy for NCD prevention and control, according to the report.

The experts found that subsidies for fresh fruit and vegetables that reduce retail prices by 10–30% increase fruit and vegetable consumption. They also reported that fiscal policies leading to a 20% increase in the retail price of sugar-sweetened beverages result in proportional reductions in consumption of such products.

The fiscal and economic policies set out in the new WHO report provide policy options for countries seeking to meet the health targets set out in the *Global action plan for the prevention and control of noncommunicable diseases 2013–2020*.

In 2014, more than 1 in 3 (39%) adults worldwide aged 18 years and older were overweight and an estimated 42 million children aged under 5 years were overweight in 2015.

http://www.who.int/dietphysicalactivity/publications/fiscal-policies-diet-prevention/

**Cover photo**

This month’s cover photo shows a fisherman who has had difficulties finding steady work since he recovered from Ebola in Freetown, Sierra Leone. There are more than 10 000 survivors of Ebola virus disease. Many of them face health problems including mental health issues. WHO issued interim guidance for public health services to support them, entitled *Clinical care for survivors of Ebola virus disease *earlier this year. http://www.who.int/csr/resources/publications/ebola/guidance-survivors/en/

**Figure:**
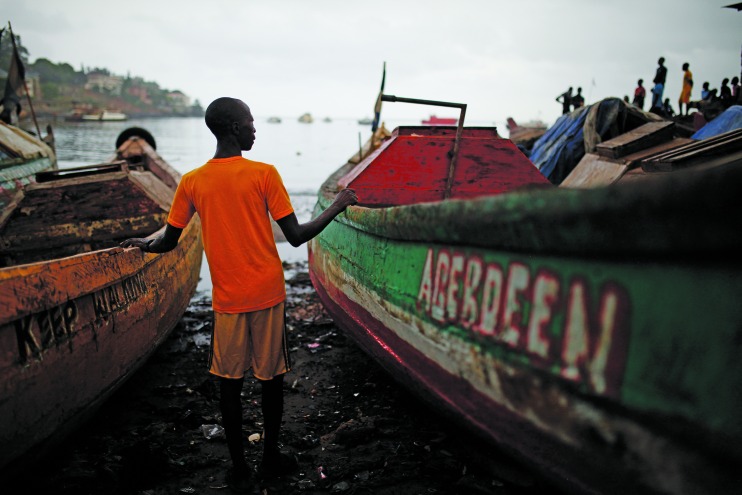
© Dominic Chavez/World Bank

## TB epidemic 

The global tuberculosis epidemic is larger than previously estimated after the inclusion of new surveillance and survey data from India, according to the *Global tuberculosis report 2016*.

According to the WHO report released last month, there were an estimated 10.4 million new (incident) tuberculosis cases worldwide in 2015 (compared with 9.6 million in 2014). Six countries accounted for 60% of the estimated 10.4 million new cases: India, Indonesia, China, Nigeria, Pakistan and South Africa.

In 2015, there were an estimated 480 000 new cases of multidrug-resistant tuberculosis (MDR–TB), which means combined resistance to isoniazid and rifampicin, and an additional 100 000 new cases that were resistant to rifampicin (RR–TB), a common first-line treatment for tuberculosis.

MDR–TB and RR–TB patients require longer treatment than those with drug–susceptible disease and in 2013 only 52% of those starting treatment for these completed it successfully.

There were an estimated 1.4 million tuberculosis deaths in 2015, and an additional 400 000 deaths among HIV-infected tuberculosis patients, the report said. Although the number of tuberculosis deaths fell by 22% between 2000 and 2015, tuberculosis remained one of the top 10 causes of death globally in 2015.

Global progress depends on major advances in tuberculosis prevention and care in the 30 countries with a high-burden of tuberculosis, as well as in those with a large MDR–TB or HIV-associated tuberculosis burdens, the report said.

High-burden countries are those with the highest estimated numbers of incident tuberculosis cases that account for 80% of the global total.

Worldwide the rate of decline in tuberculosis incidence (new cases) from 2014 to 2015 was 1.5%.

This rate will need to be accelerated to an annual decline of 4–5% by 2020 to achieve the first milestones of the End TB Strategy that was approved by the World Health Assembly in 2014.

The strategy sets targets for a 90% reduction in tuberculosis deaths and an 80% reduction in new cases between 2015 and 2030.

http://www.who.int/tb/publications/

## Health promotion event

The 9th Global Conference on Health Promotion to be held in Shanghai, China this month aims to provide guidance to WHO’s Member States on how health promotion can help them achieve the sustainable development goals (SDGs) in 2030.

The conference, entitled “Health for all and all for health”, from 21–24 November, aims to inspire governments “to grasp the great potential of promoting health across all sectors of society” as they move towards the SDGs.

A key focus of the conference is promoting urban health, and municipal leaders and mayors from around the world will present their health promotion initiatives and share their experiences.

The first global health promotion conference was held in Ottawa, Canada in 1986.

http://www.who.int/mediacentre/events/2016/health-promotion

**Looking ahead**

**14–20 November – World Antibiotic Awareness Week**

**1 December – World AIDS Day**

**23 January–1 February – 140th WHO Executive Board meeting **

